# Intimate Partner Violence During Pregnancy among Postnatal Mothers Attending Health Centers in Lalitpur District, Nepal: An Observational Study

**DOI:** 10.31729/jnma.v63i292.9264

**Published:** 2025-12-31

**Authors:** Isabel Lawot, Karuna Bajracharya, Niran Shrestha, Shreejana Singh, Deepak Raj Joshi, Jamuna Adhikari, Muna Rana Thapa, Mohan Raj Sharma

**Affiliations:** 1 Department of Midwifery, Maharajgunj Nursing Campus, Institute of Medicine, Tribhuvan University; 2 Department of Midwifery, Patan Academy of Health Science, School of Nursing and Midwifery; 3 Research Department, Institute of Medicine, Tribhuvan University; 4 Department of Community Medicine and Public Health, Maharajgunj Medical Campus, Institute of Medicine, Tribhuvan University; 5 Department of Community, Maharajgunj Nursing Campus, Institute of Medicine, Tribhuvan University; 6 Department of Neurosurgery, Institute of Medicine, Tribhuwan University, Kathmandu, Nepal

**Keywords:** *abuse*, *husband*, *intimate partner violence*, *postnatal*, *pregnancy*

## Abstract

**Introduction:**

Any form of intimate partner violence during pregnancy can push women into critical situations. It may result in inadequate prenatal care, poor nutrition, depression, and even death, which are all preventable maternal outcomes. For the neonate, the effect can be low birth weight, preterm birth, and even neonatal death. Therefore, this study aims to assess the prevalence of intimate partner violence among postnatal mothers attending Health Centres.

**Methods:**

A descriptive cross-sectional design with a probability cluster sampling technique was used for the study. The data was collected using interview schedules from 325 postnatal mothers using a modified form of the domestic violence tool from the Nepal Demographic Health Survey.

**Results:**

A total of 92 (28.30%) of mothers experienced some form of violence. Specifically, 62 (19.08%) endured psychological abuse, 49 (15.08%) suffered actual physical violence, and 52 (16.00%) were victims of sexual violence.

**Conclusions:**

This study reveals a significant prevalence of intimate partner violence, with over a quarter of postnatal mothers experiencing some form of abuse. These findings highlight a critical public health issue in Nepal, indicating that a substantial number of mothers and their infants are at risk of severe health consequences due to violence from an intimate partner.

## INTRODUCTION

Intimate partner violence (IPV) is one of the most preventable forms of violence that women experience all over the world. Physical, sexual, emotional, and controlling behaviors by an intimate partner are examples of abuse experienced by women. WHO in 2021 estimated that 30.0% of women globally experience physical or sexual violence at some point in their lives.^1^ It is consistently reported to be higher in developing countries.^1^

Various studies, such as in a study in Japan, 13.4% experienced IPV who had lower household income, low educational status and were multipara,^2^ and in an Indian study, 29.7% of pregnant women had IPV.^6^ Nepalese women with low income and lack of education for themselves and their spouses are the most common risk factors for violence during pregnancy.^4^

While IPV remains a common occurrence among Nepalese women, the majority of the existing literature has concentrated on domestic violence, leaving an absence of sufficient evidence regarding IPV. Therefore, this study aimed to assess the prevalence of intimate partner violence among postnatal mothers attending Health Centres.

## METHODS

A descriptive cross-sectional study was conducted in Lalitpur, Nepal from March to June 2022. The study focused on postnatal mothers within a year of childbirth who attended Maternal Child Health (MCH) clinics for infant immunization and postnatal checkups.

The sampling in the study used a mixed probability and nonprobability method of multi-stage cluster sampling and a convenience methodKathmandu Valley was chosen purposively, with Lalitpur selected randomly among its three districts. Again, the metropolitan city of Lalitpur was chosen randomly from the different local levels of the Lalitpur district, followed by the simple random selection of eight wards (3,4,7,8,9,12,14, and 29) using lottery method where one health centers operated from each 29 wards of the metropolitan city. Based on equal number proportion, 41 participants were taken conveniently from different clinics of each selected ward due to uncertain patient flow status.

**Figure 1:**
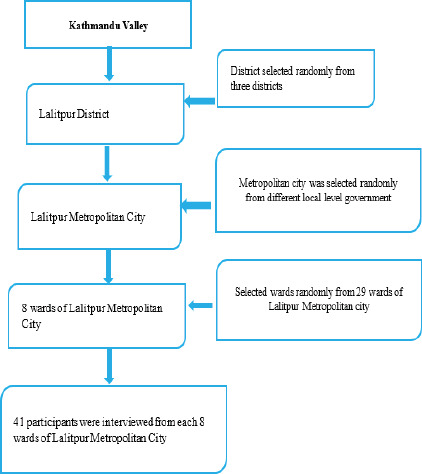
Multi-stage cluster and convenience method

The sample size was determined based on following formula: n=z^2^pq/d^2^

Where:

- n = the required sample size
- Z = the Z-score corresponding to the desired confidence level. For a 95% confidence level, which is the standard for most research, the Z-score is 1.96.
- p = the estimated prevalence of the factor. In this case, the prevalence of Intimate
- Partner Violence (IPV) from the Domestic Violence of Nepal Demographic Health Survey (NDHS) 2016 as spousal violence, which is 26% or 0.26.^6^
- d = the desired margin of error (also called precision), set at 5% or 0.05.

Adding a 10% non-response rate, the final sample size was 325. The inclusion criteria were: (a) mothers in the postnatal period up to one year, and (b) those without any major health problems such as haemorrhage, sepsis, postnatal depression, psychosis, renal/cardiac disease.

The tool used in this study contained two parts. Part I contains background information, including all the associated factors. Part II included domestic violence questions developed by the NDHS Questionnaire 2016.6. Since this tool was not originally designed to assess conditions during pregnancy, some modifications were made in this section to enhance the clarity of the subscales and to tailor the questions to the pregnancy context.

The questions include four subscales: husband’s controlling behaviour, physical, psychological, and sexual violence. Each Maternent included “Yes” and “No” response options. For items answered with “Yes,” three additional follow-up options were provided: “Often,” “Sometimes,” and “Not at all.”

There were seven items used to assess the husband’s controlling behavior during pregnancy which include try to keep you from seeing your friends; try to restrict contact with family of birth; insisted on knowing where you are all times; ignored you or treat you indifferently; get angry or jealous if you speak with Granger man; is suspicious that you are unfaithful, and expects you to ask permission before seeing health care for yourself. Psychological violence during pregnancy included 4 statements which were as mentioned below, insulted you or made you feel bad about yourself; belittled or humiliated you in front of others /publicly/family members; done things to scare you or intimidate you on purpose, eg the way he looked at you by yelling, and threatened you to hurt you or someone you care about. Physical violence during pregnancy consisted of 7 statements which were, push you, shake you or throw something at you; slap you; twist your arm or pull your hair; punch you with his fist or with something that could hurt you; kick you, drag you or beat you up; try to choke you or burn you on purpose, and threaten or attack you with a knife, gun or any other weapon. And at last, sexual violence during pregnancy comprised 3 statements such as physically forcing you to have sexual intercourse with him when you didn’t want to; having sexual intercourse you didn’t want to because you were afraid of what your husband might do, and forcing you to do something sexual acts you found degrading and humiliating.

The modified tool was evaluated by five subject matter experts for the adequacy of the content of the study questions, and a pretest was performed with 33 women from Ward 5 after the tool was translated into Nepali language or for internal consistency. The tool’s reliability was high, with a Cronbach’s Alpha score of 0.80. A structured interview schedule was performed. The Institutional Review Committee, Institute of Medicine, Tribhuvan University, Nepal, approved the study proposal (Ref number: 345/2022). The participants were interviewed. After data collection, the data were checked for consistency and entered and analysed using SPSS version 16. The percentages and frequencies were used for descriptive statistics.

## RESULTS

The study comprised a sample of 325 postnatal mothers, the vast majority of whom were 21 years or older 300 (92.30%). The mean and standard deviation of age of the participants DECEMBER 2025 was 28.21±5.743. Participants were predominantly Hindu 251 (77.23%) and identified their primary occupation as homemakers 229 (70.46%). A majority had attained a secondary or higher level of education 205 (63.08%) and resided in a nuclear family structure 196 (60.30%). Notably, health-seeking behaviors were excellent, with nearly all participants reporting antenatal clinic attendance 298 (91.69%) and institutional delivery 304 (93.54%).

**Table 1:** Demographic and Obstetric information of the participants(n=325).

Variables	n(%)
**Age (years)**	
≤ 20	25(7.70)
≥ 21	300(92.30)
**Religion**	
Hindu	251(77.23)
Other than Hindu	74(22.77)
**Ethnicity**	
Janjati	197(60.61)
Others	128(39.39)
**Education**	
Primary and below	120(36.92)
Secondary and above	205(63.08)
**Occupation**	
Homemaker	229(70.46)
Other than homemaker	96(29.54)
**Type of Family**	
Nuclear	196(60.30)
Joint	129(39.70)
**Age at Marriage (years)**	
≤ 20	149(45.85)
≥ 21	176(54.15)
**Type of Marriage**	
Love or Elope	178(54.77)
Arranged	147(45.23)
**Number of Children**	
One	159(48.92)
Two or more	176(51.08)
Abortion/Miscarriage	46(14.15)
Antenatal Clinic Visit	298(91.69)
**Place of Last Delivery**	
Health institute	304(93.54)
Home	21(6.46)
**Decision Making in Family**	
Husband only	110(33.85)
Herself or in-laws	251(66.15)

Overall, intimate partner violence was experienced by 92 (28.30%) of participants. Among them, 74 (22.77%) controlling behaviours, 62 (19.08%) psychological violence, 49 (15.08%) physical violence and 52 (16.00%) sexual violence, respectively.

**Table 2:** Prevalence of overall and different types of intimate partner violence (n=325).

Type of Violence	n(%)
Overall IPV	92(28.30)
Controlling behavior	74(22.77)
Psychological violence	62(19.08)
Physical violence	49(15.08)
Sexual violence	52(16.00)

## DISCUSSION

In this study, the intimate partner violence was assessed among 325 postnatal mothers who attended the MCH clinics of Lalitpur Metropolitan City. Among them, 7.70 % were less than equal to 20 years of age, and the mean age of the participants was 28.2121 ±5.74. This study is similar to a study conducted in Brazil, which shows the psychological and physical violence by intimate partners were 26.8% and 12.2% respectively.^7^ This study was not aligned with the study conducted in Nepal; the participants aged from 14 to 24 were 42.2% and psychological violence remained associated with age.^8^ Similarly, a study in India shows that age and IPV have a significant association with IPV, which means the lower the age, experience higher the experience of violence.^9^ Regarding education, 63.08 % of participants had a secondary level of education, and 70.46 % were homemakers; however, the study done in Nigeria shows the education level of participants was illiteracy (64.1%), 87.9% housewives who had higher IPV.^10^ This suggests that education and occupation can play an important role in intimate partner violence status.

The likelihood of experiencing violence can be instuenced by demographic factors, type of marriage, number of children, history of abortion, place of last delivery, and level of decisionmaking power. In the present study, 54.85% of participants had a love or elope marriage, 51.08% had more than two children, 14.15% reported a history of abortion, 6.46% delivered their last child at home, and 33.85% lacked the freedom to make decisions independently. A qualitative study by Deuba et al. revealed that there are different causative factors of IPV, such as marriage at a young age, refusal to have sex with their partner, having a girl child, identification of a female fetus by USG before childbirth, and alcohol consumption by the partner.^11^

The findings of the study disclosed that 8.31% of participants did not visit the antenatal clinic for their pregnancy checkup, whereas a study in Nepal found that emotional and physical violence combined with alcohol-associated less or avoided ANC visits.^8^ This finding suggests that intimate partner violence (IPV) may hinder women from attending antenatal care (ANC) visits, potentially endangering the health of both the mother and the baby. The place of delivery in the present study was reported as home (6.46%), though the study reported that women who were exposed to physical violence were significantly more likely to report that they did not use of health institute for delivery.^8^ Recent physical and sexual IPV were both linked to a lower rate of institutional birth and leaving delivery facilities earlier.^6^ The study shows that participants have two or more children (51.08 %), which is aligned with the study conducted in India, which found that lack of education, poverty, having three or more children, and dwelling in rural areas are at greater risk of being victims of violence.^23^ IPV can make a more critical situation for mothers and newborns, as the maternal period is a normal physiological process, but it is a high-risk state of life as well.^7^ IPV during pregnancy and the effect of IPV is significantly associated with fewer antenatal visits.^11^

The findings of the study revealed that overall, 28.30% of participants experienced any type of IPV during pregnancy, which is near to a study conducted in Ethiopia shows that 40.8% of participants experienced IPV during their pregnancy^12^- whereas a study conducted in Nepal shows a slightly less that Ethiopia in prevalence of intimate partner violence, which is 28.90%.^13^ This may be the study conducted in the same country. Similarly, national-level data on IPV (26.00 %) is also in line with the current study findings.^6^ The current study identified the prevalence of different types of violence, such as 15.08% Physical, 19.08% psychological, 16.00% sexual violence, and 22.77% had husband controlling behavior, however, in Bangladesh, the prevalence of IPV is around 45.29%, with 44.12% emotionally victimised, 15.29% physically abused, 10.59% sexually abused, and 19.22% abused both physically and sexually.^14^ A similar study shows almost the same prevalence, which is 16.10% psychological, 7.60% physical, and 2.70%sexual violence.^7^ Similarly, the study findings show that 27.20% of women have been subjected to some sort of violence from their husbands, with sexual violence (17.30%), psychological violence (16.60%), and physical violence (3.20%) accounting for the majority of violence.^15^ These evidences advocate that the intimate partner violence is prevalent in a broad range of regions in different forms, which may affect the overall health of the mother, baby and family as well. Inadequate prenatal care, poor nutrition, depression, and even death are all preventable maternal and neonatal outcomes; nevertheless, for neonates, there are low birth weight, preterm birth, and neonatal death.^16^ Physical aggression is directly experienced by women .^17^

Controlling behaviour is also a form of violence, which is shown in 22.77% in this study, however, a study in Nepal shows that the associated factors of domestic violence included the ethnic culture of Janjati ethnicity, illiteracy of the women, and a husband who behaved in a controlling manner.^18^ Controlling behaviour was the most common type of IPV during pregnancy (20.20%), followed by emotional (18.60%), sexual (10.60%), economic (6.10%), and physical violence (5.30%),^19^ which is still high because it is a violation of the human right to live freely. It is supported by the study findings, which state that controlling behaviors by an intimate partner is also the most common type of abuse experienced by women.^20^ Though the Nepal Demographic Health Survey NDHS 2011 reported that almost one-third of women experienced any form of violence from their partners,^21^ which decreased in 2016, 26%.^6^ In a patriarchal nation like Nepal, male family members frequently govern women’s lives. Husbands’ attempts to tightly regulate and monitor their wives’ behavior may be another manifestation of women’s subservient role in the household.^22^ A similar situation was seen in Bangladesh, where, in comparison to spouses, women were more likely to encounter IPV if they lived in food-insecure households, had poor socioeconomic positions, had little autonomy, or experienced educational inequalities.^8^

The study had certain limitations. It included only postnatal mothers within one year after childbirth, which may have introduced recall bias and instuenced the findings. Additionally, participants could not be selected through simple random sampling because a sampling frame was not available in the outpatient department.

## CONCLUSION

This study reveals that a substantial portion of postnatal mother’s experience intimate partner violence, a finding that is alarmingly consistent with broader national survey data and confirms IPV as a persistent public health issue. Controlling behaviour was the most frequently reported form of abuse, followed by psychological, sexual, and physical violence. The high prevalence of IPV among this vulnerable population has serious implications for both maternal and child health, linking to adverse outcomes like reduced antenatal care and poor neonatal health. Therefore, it is crucial to integrate IPV screening and support services into routine Maternal and Child Health clinics, where providers can identify at-risk mothers and offer confidential support. Addressing the root causes of IPV through targeted community interventions is essential to protect the well-being of mothers and their children in Nepal.

## Data Availability

The data are available from the corresponding author upon reasonable request.
